# Increased Risk of Cardiovascular Diseases in Patients With Chronic Hypoparathyroidism in Sweden

**DOI:** 10.1210/clinem/dgaf257

**Published:** 2025-04-28

**Authors:** Sigridur Björnsdottir, Michael Mannstadt, Bart Clarke, Tim Spelman, Olle Kämpe, Gianluigi Savarese

**Affiliations:** Department of Molecular Medicine and Surgery, Karolinska Institutet, 171 77 Stockholm, Sweden; Endocrine Unit, Massachusetts General Hospital and Harvard Medical School, Boston, MA 02114, USA; Division of Endocrinology, Diabetes, Metabolism, and Nutrition, Mayo Clinic, Rochester, MN 55905, USA; Department of Clinical Neuroscience, Karolinska Institutet, 171 77 Stockholm, Sweden; Department of Medicine, Solna, Center for Molecular Medicine, Karolinska Institutet, 171 76 Stockholm, Sweden; Department of Endocrinology, Metabolism and Diabetes, Karolinska University Hospital, 171 76 Stockholm, Sweden; Division of Cardiology, Department of Medicine, Karolinska Institutet, 171 77 Stockholm, Sweden; Heart and Vascular and Neuro Theme, Karolinska University Hospital, 171 76 Stockholm, Sweden

**Keywords:** hypoparathyroidism, cardiovascular diseases, epidemiology

## Abstract

**Context:**

Data on cardiovascular outcomes in patients with chronic hypoparathyroidism (hypoPT) are limited.

**Objective:**

To investigate the risk of cardiovascular outcomes, acute myocardial infarction, atrial fibrillation/flutter, heart failure, valvular heart disease, peripheral artery disease, and stroke/transient ischemic attack (TIA) in patients with chronic hypoPT.

**Design:**

The Swedish National Patient Registry, the Swedish Prescribed Drug Registry, and the Total Population Registry, 1997-2018.

**Settings:**

Population-based cohort study in Sweden.

**Patients:**

National registries were used to identify patients with chronic hypoPT and matched controls.

**Results:**

A total of 1982 with chronic hypoPT and 19 499 controls were included. After adjustment for cardiovascular risk factors, patients with chronic hypoPT had higher risk of valvular heart disease [hazard ratio (HR) 2.08; 95% confidence interval (CI) 1.67-2.60], peripheral artery disease (HR 1.78; 95% CI 1.41-2.26), heart failure (HR 1.66; 95% CI 1.44-1.90), atrial fibrillation/flutter (HR 1.58; 95% CI 1.38-1.81), acute myocardial infarction (HR 1.31; 95% CI 1.05-1.64), and fatal cardiovascular disease (HR 1.59; 95% CI 1.40-1.80) compared to matched controls. No significant difference in risk of stroke/TIA was observed. Cardiovascular outcomes did not differ between patients with surgical and nonsurgical chronic hypoPT. Females with hypoPT had a significantly increased risk of valvular heart disease, peripheral artery disease, heart failure, atrial fibrillation, myocardial infarction, and fatal cardiovascular disease compared to female controls. There were no differences in any cardiovascular outcomes between males with hypoPT and male controls.

**Conclusion:**

The risk of cardiovascular diseases was increased in patients with chronic hypoPT, particularly among women. These findings highlight the need for close monitoring and preventive management of cardiovascular risk factors, especially in women.

Chronic hypoparathyroidism (hypoPT) is a rare disorder, most commonly caused by anterior neck surgery, which accounts for 75% of cases (1). Other etiologies include genetic disorders, autoimmune destruction of the parathyroid glands, infiltrative disorders, and exposure to ionizing radiation. HypoPT is defined by low serum calcium levels and an inappropriately low production of PTH. The prevalence of chronic hypoPT is estimated to range between 6.4 and 37 per 100 000 individuals across different countries (2-5). The mainstay of conventional treatment for patients with chronic hypoPT is activated vitamin D and oral calcium supplement aimed at correcting the hypocalcemia. However, this treatment does not restore the full spectrum of PTH's physiological functions (1). Recently, replacement with PTH, the lacking hormone, has been approved as a new treatment option (6).

Several studies have highlighted an increased risk of cardiovascular diseases in patients with chronic hypoPT (7-9). However, due to the rarity of hypoPT, data on potential cardiac and vascular complications remain limited. Existing evidence is primarily derived from small register-based studies or retrospective case-control analyses, which often lack sufficient statistical power to detect clinically significant associations after appropriate adjustments. As a result, findings on cardiovascular outcomes in patients with nonsurgical and postsurgical hypoPT have been inconsistence (7, 8, 10, 11). Therefore, additional evidence is required. By merging data from population-based registers in Sweden, we were able to identify a large cohort of patients with chronic hypoPT receiving conventional treatment. This enabled us to investigate the association between chronic hypoPT and cardiovascular outcomes, as well as mortality, in a large cohort compared to matched controls.

## Subjects and Methods

### Registries

The Swedish National Patient Registry (SNPR) includes inpatient data since 1964, with nationwide coverage since 1987 and hospital outpatient data since 2001. While it does not capture data from primary care settings, most patients with chronic hypoPT in Sweden are managed through hospital outpatient clinics. The SNPR includes information on age, sex, dates of hospital admission and discharge, surgical procedures codes, and discharge diagnoses. Reporting to the SNPR is mandatory for all healthcare providers (12). Data from the SNPR can be linked to other registries through the unique personal identity number assigned to all Swedish residents.

The Swedish Prescribed Drug Registry (SPDR) has collected data on all prescription drugs dispensed to the Swedish population since July 2005 (13). It includes data on prescriptions from primary care and classifies medications using the Anatomical Therapeutic Chemical (ATC) classification system. Registration of all prescribed and dispensed drugs is mandatory for pharmacies in Sweden. However, the SPDR does not cover over-the-counter medications, drugs used in hospital settings, or clinical information on diagnoses or treatment indications.

The Cause of Death Registry has maintained records since 1961 for all individuals who were registered in Sweden at the time of their death. It includes information on both the underlying and immediate causes of death, with diagnoses coded according to the International Classification of Diseases (ICD) and a coverage rate exceeding 99.5% (14).

Additionally, the Swedish Total Population Registry collects demographic data on the country's population (15).

### Participants

This registry-based study was approved by the Research Ethics Board of Stockholm, Sweden (approval no. DNR: 2017/476-31/4). We used the SNPR to identify individuals with the ICD-10 diagnosis of hypoPT (ICD-10: E20.0, E20.2-9), postsurgical hypoPT (ICD-10: E89.2), DiGeorge syndrome (ICD-10 D82.1), and autoimmune polyglandular failure (ICD-10: E31.0) between January 1997 and December 2018. This interval was chosen as specific ICD-10 codes for hypoPT were introduced in 1997. The SPDR that started in 2005 was used to enhance the diagnostic accuracy by including only patients with at least 2 dispensations of active vitamin D [dihydrotachysterol (ATC A11CC02), alfacalcidol (ATC A11CC03), or calcitriol (ATC A11CC04)] with or without calcium supplements. For those diagnosed from 2005 and onward, these dispensations were required at least 13 months postdiagnosis. For those diagnosed before 2005, at least 2 dispensations of active vitamin D were required within the first year of SPDR. This approach ensured the inclusion of patients with chronic, not transient, hypoPT.

Patients were excluded if they had fewer than 2 dispensations of active vitamin D during the final year of follow-up. To further improve the diagnosis precision, we excluded patients and their controls with kidney failure at baseline or within 1 year from baseline (ICD codes provided in Supplementary Table S1) (16), as these individuals are often treated with active vitamin D for secondary hyperparathyroidism and may have been incorrectly coded for hypoPT.

**Figure 1.**
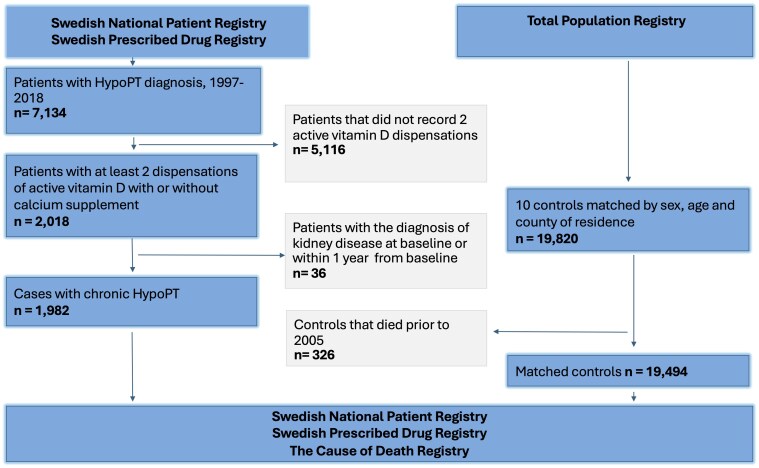
Flowchart of study participants.

For each patient, we randomly identified 10 controls matched by year of birth, sex, and county of residence using the Total Population Registry including all Swedish residents alive at the end of each year (Fig. 1). Matched controls who died prior to 2005 were excluded to avoid immortal time bias. The follow-up for cases and matched controls began 13 months after the first recorded hypoPT diagnosis in the SNPR for individuals diagnosed in 2005 or later. For those with an initial hypoPT diagnosis in SNPR prior to 2005, follow-up started at the time of the second dispensation of active vitamin D in the drug registry. Follow-up ended on December 31, 2018, or on the last recorded activity in the registries, whichever came first.

The accuracy of similar diagnostic criteria for identifying patients with chronic hypoPT in Sweden has been validated through medical chart reviews in a previous study (17), confirming the diagnosis in 91% of 120 randomly selected cases.

### Outcome Measures and Covariates

A cardiovascular event was defined as the presence of at least 1 diagnosis code for acute myocardial infarction (ICD-10: I21, I22, I25.2), atrial fibrillation/flutter (ICD-10: I48), heart failure (ICD10: I11.0, I13.0, I13.2, I25.5, I42.0, I42.3, I42.5-9, I43, I50, J81, K76.1, R57.0), valvular heart disease (ICD-10: I05-I08, I34-I39), peripheral artery disease (ICD-10: I70-73), and stroke/transient ischemic attack (TIA) (ICD-10: I60-64, I69, G45) identified through the SNPR. Fatal cardiovascular disease was defined as death attributed from a cardiovascular causes, including diseases of the circulatory system (ICD-10: I), chronic congestion of the liver (ICD-10: K76.1), pulmonary edema (ICD-10: J81), transient cerebral ischemic attack (ICD-10: G45), and cardiogenic shock (ICD-10: R57), as recorded in the Cause of Death Registry.

Comorbidities were defined as follows: hypertension was identified in the presence of ICD-10 code I10-15 and/or multiple (≥2) dispensations of antihypertensive medications, including antihypertensives (ATC C02), diuretic (ATC C03), β-blockers, (ATC C07), calcium channel blockers (ATC C08), and agents acting on the renin-angiotensin system (ATC C09) through SPDR. Dyslipidemia was defined by ICD-10 code E78 and/or multiple (≥2) prescriptions of lipid-modifying agents (ATC C10). Type 1 diabetes was identified by ICD-10 code E10 and type 2 diabetes by ICD-10 code E11 and/or multiple (≥2) prescriptions of antidiabetic agents (ATC A10). Chronic obstructive pulmonary disease (COPD) defined by ICD-10 code J44 was used as a proxy for heavy smoking. Additionally, thyroxin use (ATC H03AA01) was analyzed in patients with chronic hypoPT and matched controls through SPDR.

### Statistical Analysis

Categorical variables were summarized using frequency and percentage. Continuous variables were summarized using mean and SD or median and interquartile range (IQR) as appropriate. A McNemar chi-square test was used to compare risk factors at baseline between cases and their matched controls. Multivariable marginal Cox regression models were used to estimate the adjusted hazard ratios (HRs) with 95% confidence intervals (CIs) for the associations between incident cardiovascular disease and chronic hypoPT. Adjustments were made for the following risk factors for cardiovascular disease: history of diabetes, hypertension, hyperlipidemia, COPD (as a proxy for heavy smoking), thyroxine use, acute myocardial infarction, atrial fibrillation, heart failure, valvular heart disease, peripheral artery disease, and stroke/TIA. Hazard proportionality was assessed via analysis of scaled Schoenfeld residuals. An interaction term between sex, surgical cause, and nonsurgical cause and hypoPT was separately included in the multivariable Cox regression models to identify whether the association between hypoPT and outcomes varied across different disease etiology subgroups. We conducted a subgroup analysis to compare the risk of cardiovascular diseases separately among females and males compared to controls. Additionally, a multivariable analysis was performed to identify significant demographic and clinical risk factors associated with cardiovascular outcomes among patients with chronic hypoPT.

For all analyses, a *P* < .05 was considered statistically significant. All analyses were conducted using Stata version 18 (StataCorp, 2023, Stata Statistical Software: Release 18, College Station, TX) and R version 4.0.5 (R Foundation for Statistical Computing, Vienna, Austria).

## Results

### Patient Characteristics

A total of 1982 patients with chronic hypoPT and 19 494 matched controls were alive at the start of follow-up and included in the study. Among patients with chronic hypoPT, 76.7% were women, and the mean age (SD) at inclusion was 54.7 (19.7) years. Of these, 71.8% had postsurgical hypoPT, while 28.2% had nonsurgical hypoPT. The median follow-up time was 9.09 years (IQR 4.34-14.63) for patients with chronic hypoPT and 8.91 years (IQR 4.27-14.38) for controls (Table 1).

**Table 1.** Baseline characteristics of patients with chronic hypoPT and matched controls in Sweden

	Chronic hypoPT	Controls	*P*-value
No. of patients	1982	19 494	
Age (years), mean (SD)	54.7 (19.7)	54.7 (19.6)	.989
Women, n (%)	1520 (76.7)	14 942 (76.7)	.967
Follow-up time, years (median, IQR)	9.09 (4.34-14.63)	8.91 (4.27-14.38)	.887
Outpatient visits per year (median, IQR)	3.51 (1.99-6.12)	NA	
Etiology			
Postsurgical hypoPT	1423 (71.8)	NA	
DiGeorge syndrome	27 (1.4)	NA	
Idiopathic hypoPT	245 (12.4)	NA	
Other or unspecified hypoPT	265 (13.4)	NA	
Dispensations of active vitamin D per year*^a^*	6.2 (4.3-10.2)	NA	
Dispensations of calcium supplementation per year	3.4 (1.2-6.3)	NA	
Hypertension	682 (34.4)	3797 (20.4)	**<**.**001**
Dyslipidemia	217 (11.0)	1891 (9.7)	.075
Diabetes			
Type 1 diabetes	63 (3.2)	278 (1.4)	**<**.**001**
Type 2 diabetes	138 (7.0)	895 (4.6)	**<**.**001**
Chronic obstructive pulmonary disease	151 (7.6)	732 (3.8)	**<**.**001**
Thyroxine use	892 (45.0)	898 (4.6)	**<**.**001**
Previous cardiovascular disease, before start of follow-up			
Ischemic heart disease	136 (6.9)	913 (4.7)	**<**.**001**
Acute myocardial infarction	61 (3.1)	377 (1.9)	.**001**
Stroke/TIA	100 (5.1)	636 (3.3)	**<**.**001**
Atrial fibrillation/flutter	130 (6.6)	603 (3.1)	**<**.**001**
Heart failure	100 (5.1)	426 (2.2)	**<**.**001**
Valvular heart disease	47 (2.4)	201 (1.0)	**<**.**001**
Peripheral artery disease	34 (1.7)	208 (1.1)	.**009**

Values for continuous data are mean ± SD or median (IQR) and count (%) for categorical data. International Classification of Diseases codes of etiology: postsurgical hypoparathyroidism (E89.2), DiGeorge syndrome (D82.1), autoimmune polyendocrine syndrome (E31.0), idiopathic hypoparathyroidism (E20.0), other or unspecified hypoparathyroidism (E20.2–9). Bold values indicate statistically significant associations (*P* < .05).

Abbreviations: hypoPT, hypoparathyroidism; IQR, interquartile range; NA, not applicable; TIA, transient ischemic attack.

^*a*^Dispensation of any of the Anatomical Therapeutic Chemical codes A11CC02, A11CC03, A11CC04.

At the start of follow-up, patients with chronic hypoPT were more likely than controls to have hypertension, type 1 and 2 diabetes, COPD, and a history of cardiovascular diseases (Table 1).

### Outcome Analysis

**Figure 2.**
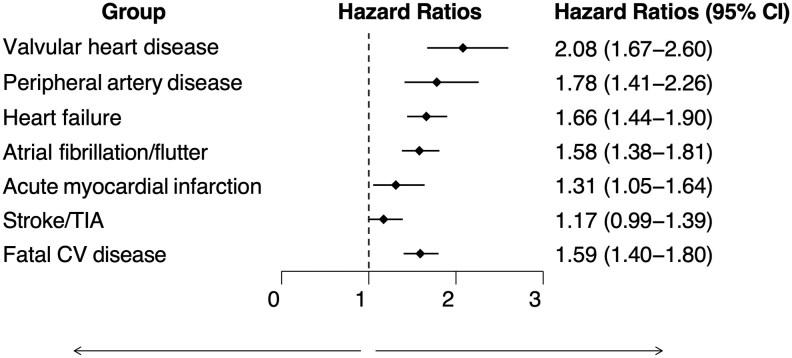
Adjusted HRs for cardiovascular outcomes in patients with chronic hypoparathyroidism compared to matched controls. Adjusted for diabetes, hypertension, hyperlipidemia, chronic obstructive pulmonary disease, thyroxine use, and history of (before start of follow-up) any of the cardiovascular disease outcomes at baseline listed in Table 1. Abbreviations: CI, confidence interval; HR, hazard ratio; PY, person-years; TIA, transient ischemic attack.

Patients with chronic hypoPT demonstrated a higher crude rate of several cardiovascular conditions compared to controls, including valvular heart disease (HR 2.12; 95% CI 1.74-2.59), heart failure (HR 1.78; 95% CI 1.57-2.02), peripheral artery disease (HR 1.73; 95% CI 1.39-2.15), atrial fibrillation/flutter (HR 1.68; 95% CI 1.48-1.90), acute myocardial infarction (HR 1.29; 95% CI 1.05-1.69), and stroke/TIA (HR 1.24; 95% CI 1.06-1.45) (Table 2). These associations persisted after adjustment for diabetes, hypertension, hyperlipidemia, COPD (as a proxy for smoking), thyroxine use, and baseline cardiovascular disease history, except for stroke/TIA (Fig. 2).

**Table 2.** Crude HRs for cardiovascular outcomes in patients with chronic hypoPT compared to matched controls

Outcome	Chronic hypoPT (n = 1982)		Controls (n = 19 494)		Unadjusted HR	95% CI
	Events, n	Events/1000 PY (95% CI)	Events, n	Events/1000 PY (95% CI)		
Valvular heart disease	116	5.98 (4.98-7.17)	559	2.81 (2.58-3.05)	**2**.**12**	1.74-2.59
Peripheral artery disease	95	4.89 (4.00-5.98)	560	2.81 (2.58-3.05)	**1**.**73**	1.39-2.15
Heart failure	279	14.80 (13.16-16.64)	1609	8.24 (7.85-8.66)	**1**.**78**	1.57-2.02
Atrial fibrillation/flutter	299	16.25 (14.51-18.21)	1845	9.57 (9.15-10.02)	**1**.**68**	1.48-1.90
Acute myocardial infarction	99	5.08 (4.17-6.19)	776	3.91 (3.65-4.20)	**1**.**29**	1.05-1.69
Stroke/TIA	172	9.01 (7.76-10.46)	1409	7.24 (6.87-7.62)	**1**.**24**	1.06-1.45
Fatal cardiovascular disease	347	13.00 (11.70-14.44)	2571	9.55 (9.19-9.93)	**1**.**40**	1.25-1.57

Bold values indicate statistically significant associations (*P* < .05).

Abbreviations: CI, confidence interval; HR, hazard ratio; hypoPT, hypoparathyroidism; PY, person-years; TIA, transient ischemic attack.

Additionally, patients with chronic hypoPT had both higher crude (HR 1.40; 95% CI 1.25-1.57) and adjusted (HR 1.59; 95% CI 1.40-1.80) rates of a fatal cardiovascular disease compared to controls (Fig. 2). Results remained consistent after excluding patients with prebaseline thyroid cancer (data not shown).

No statistically significant difference was observed in the risk of any cardiovascular outcome between surgical vs nonsurgical chronic hypoPT (Table 3).

**Table 3.** Interaction testing stratified by sex and surgical and nonsurgical chronic hypoPT

Outcome	Female compared to male *P*-value	Surgical- compared to nonsurgical chronic hypo PT *P*-value
Atrial fibrillation/flutter	<.001	.664
Myocardial infarction	.140	.410
Heart Failure	.029	.242
Stroke/TIA	.939	.859
Valvular heart disease	.006	.309
Peripheral artery disease	.007	.806
Cardiovascular death	.510	.299

Abbreviations: hypoPT, hypoparathyroidism; TIA, transient ischemic attack.

Women with chronic hypoPT had a higher risk of atrial fibrillation/flutter, valvular heart disease, peripheral artery disease, and heart failure compared to men (Table 3). However, there was no significant differences in cardiovascular death between female and male patients with chronic hypoPT (*P* = .510).

### Subgroup Analysis

In a subgroup analysis restricted to female participants, chronic hypoPT was significantly associated with an increased risk of several cardiovascular outcomes compared with female controls, including valvular heart disease (HR 2.43; 95% CI, 1.90-3.12), peripheral artery disease (HR 2.20; 95% CI, 1.68-2.29), heart failure (HR 1.83; 95% CI, 1.56-2.15), atrial fibrillation (HR 1.81; 95% CI, 1.55-2.10), myocardial infarction (HR 1.42; 95% CI, 1.09-1.86), and fatal cardiovascular disease (HR 1.25; 95% CI, 1.09-1.44), after adjustment for baseline confounders. No significant difference was observed in the risk of stroke or TIA between female cases and controls. In contrast, no significant differences in any cardiovascular outcomes were found between males with chronic hypoPT and their matched male controls (Table 4).

**Table 4.** Adjusted*^a^* hazard ratios and *P*-values for cardiovascular outcomes among females and males with chronic hypoPT compared to their matched controls

Outcomes	Females Cases vs controls		Males Cases vs controls	
	Hazard Ratio (95% CI)	*P*-value	Hazard ratio (95% CI)	*P*-value
Valvular heart disease	**2.43** (**1.90, 3.12)**	**<**.**001**	1.07 (0.65, 1.78)	.787
Peripheral artery disease	**2.20** (**1.68, 2.89)**	**<**.**001**	0.92 (0.55, 1.56)	.770
Heart failure	**1.83** (**1.56, 2.15)**	**<**.**001**	1.18 (0.88, 1.58)	.270
Atrial fibrillation/flutter	**1.81** (**1.55, 2.10)**	**<**.**001**	0.91 (0.68, 1.22)	.541
Acute myocardial infarction	**1.42** (**1.09, 1.86)**	.**009**	1.03 (0.67, 1.58)	.894
Stroke/TIA	1.15 (0.94, 1.40)	.167	1.13 (0.80, 1.58)	.483
Fatal cardiovascular disease	**1.25** (**1.09, 1.44)**	.**002**	1.19 (0.92, 1.54)	.175

Bold values indicate statistically significant associations (*P* < .05).

Abbreviations: CI, confidence interval; hypoPT, hypoparathyroidism.

^*a*^Adjusted for baseline comorbidities and age.

In a multivariable analysis of key predictors for cardiovascular outcomes among patients with chronic hypoPT, older age at baseline was associated with an increased risk of all cardiovascular outcomes (Supplementary Table S2) (16). Type 1 diabetes was linked to a higher risk of peripheral artery disease and myocardial infarction. Hypertension was associated with increased risks of peripheral artery disease, atrial fibrillation, and stroke. COPD was linked to elevated risks of valvular heart disease, heart failure, atrial fibrillation, and myocardial infarction. Older age, female sex, surgical etiology, and type 2 diabetes were associated with increased risk of fatal cardiovascular disease. Prior thyroxine use was associated with a reduced risk of all cardiovascular outcomes.

## Discussion

Our study showed that patients with chronic hypoPT had an increased risk of valvular heart disease, peripheral artery disease, heart failure, atrial fibrillation/flutter, and acute myocardial infarction even after adjusting for cardiovascular risk factors. Importantly, the risk of fatal cardiovascular disease was approximately 59% higher in patients with chronic hypoPT compared to controls. No significant difference was observed in the risk of stroke/TIA between the 2 groups. The increased cardiovascular risk associated with chronic hypoPT was consistent regardless of the underlying etiology. Furthermore, women with chronic hypoPT exhibited a higher risk of atrial fibrillation/flutter, valvular heart disease, peripheral artery disease, and heart failure compared to men with chronic hypoPT.

In our study, patients with chronic hypoPT had higher cardiovascular risk factors at baseline compared to controls. This difference may be attributed to detection bias, as patients with hypoPT are more likely to engage with a healthcare provider than controls. Despite this, the increased cardiovascular risk persisted even after adjusting for known cardiovascular risk factors.

Previous studies on cardiovascular complications in patients with chronic hypoPT have reported various results based on etiology. Two studies focusing on nonsurgical chronic hypoPT patients showed increased cardiovascular risk. A Danish study of 180 patients found a nearly 2-fold increased risk of ischemic heart disease, arrhythmia, and stroke (11), while a Korean study of 897 patients reported a higher risk of arrhythmia and heart failure (18). Neither study observed increased mortality.

The same group from Denmark found no increased risk of cardiovascular diseases in 688 patients with postsurgical hypoPT compared to controls (8). In contrast, a UK population-based study of 280 patients with surgical and nonsurgical hypoPT reported increased risk of cardiovascular diseases but not in cerebrovascular risk (10). A large US study using insurance claims data also found higher cardiovascular and cerebrovascular risk in patients with chronic hypoPT, but the control group was on average 11 years younger (7).

The potential difference in cardiovascular outcomes between surgical and nonsurgical hypoPT remains debatable, with questions about whether these differences are pathophysiological or related to disease duration. Our study demonstrates an increased risk of cardiovascular events in both surgical and nonsurgical hypoPT compared to controls, differing from earlier findings. This may be due to our larger population of surgical hypoPT patients and longer follow-up, which provided greater statistical power.

The pathophysiological basis for the increased risk of cardiovascular diseases in patients with chronic hypoPT is not fully understood. Possible mechanisms include fluctuations in calcium levels, hyperphosphatemia, and a lack of PTH. A US case-control study linked higher cardiovascular risk to higher serum calcium and phosphate levels outside the normal ranges (19), while a Danish study associated it with lower ionized calcium levels, more hypercalcemic episodes, and longer disease duration (9). Interestingly, hypocalcemia, rather than hypercalcemia, was more strongly linked to increased cardiovascular risk in this study, possibly due to its known effects on QT-interval prolongation and arrhythmias (20).

Calcium fluctuation may also explain the nearly 2-fold increased risk of peripheral artery disease in our cohort. A Danish hospital-based study found increased lower extremities arterial calcifications in patients with hypoPT, linked to higher serum calcium and lower glomerular filtration rates (21). Similarly, hyperphosphatemia was associated with cardiac valve calcification in a Brazilian study, while calcium and vitamin D levels were not (22). A Turkish study reported increased arterial stiffness and blood pressure in patients with hypoPT, with hyperphosphatemia and advanced age as the key contributors (23). Increased arterial stiffness, measured by pulse wave velocity, is a strong predictor of cardiovascular events. These findings suggest that improved biochemical control in chronic hypoPT may help mitigate cardiovascular risk.

PTH receptors are present in endothelial and myocardial cells, with PTH shown to have direct hypertrophic effects on myocardial cells (20). As a potent vasodilator, PTH primarily affects small distal resistance vessels or arterioles rather than large arteries like the aorta (24). Loss of PTH action on vascular smooth muscle, endothelial cells, cardiomyocytes, and the cardiac conducting system may contribute to cardiovascular complications in chronic hypoPT (25). A recent study of 113 patients with chronic hypoPT treated with recombinant human PTH (1-84) compared with a historical control cohort of patients not treated with recombinant human PTH (1-84) found a lower risk of cardiovascular conditions over 5 years compared to those on conventional therapy (26). Further research is needed to determine whether PTH treatment offers long-term cardiovascular benefits by improving mineral homeostasis.

The reasons behind the higher cardiovascular risk observed in women compared to men with chronic hypoPT remain unclear. It may reflect sex-specific differences in mineral metabolism, hormonal regulation, or cardiovascular susceptibility. Estrogen deficiency has been linked to endothelial dysfunction and vascular calcification, which may exacerbate hypoPT-related risks in women (27, 28). Additionally, females may experience more significant disturbances in calcium-phosphate balance or differences in treatment response. The absence of increased cardiovascular risk among males may either reflect a true biological difference or be influenced by limited statistical power due to smaller sample sizes. Further studies are warranted to better understand the mechanisms driving these sex-specific associations.

In our cohort, cardiovascular events were more frequently fatal in the chronic hypoPT group than in controls, potentially indicating greater disease severity. Further research is needed to explore the mechanism linking chronic hypoPT to cardiovascular risk.

The strengths of this study include the use of a large cohort from high-quality nationwide registries and data linkage to combine ICD codes with prescription drug records to identify patients with chronic hypoPT. Furthermore, ICD-10 codes for hypoPT have been previously validated, showing a positive predictive value of 91% (17). However, the study has several limitations. We lacked data on biochemical measures, physical activity, height and body weight, blood pressure, and smoking habits, relying instead on COPD as a proxy for heavy smoking. These factors could serve as potential time-varying confounders in the relationship between chronic hypoPT and cardiovascular diseases. Additionally, doses of active vitamin D, calcium, or L-thyroxine were not available in the registries.

## Conclusion

Using a large, population-based cohort, we identified an increased risk of cardiovascular diseases and cardiovascular mortality in women with chronic hypoPT in Sweden compared to women without hypoPT. These findings highlight the need for close monitoring and preventive management of cardiovascular risk factors, particularly in women.

## Data Availability

Some or all datasets generated during and/or analyzed during the current study are not publicly available but are available from the corresponding author on reasonable request.

## Abbreviations

CI: confidence interval
COPD: chronic obstructive pulmonary disease
HR: hazard ratio
hypoPT: hypoparathyroidism
IQR: interquartile range
SNPR: Swedish National Patient Registry
SPDR: Swedish Prescribed Drug Registry
TIA: transient ischemic attack
